# The influence of brand marketing on consumers’ emotion in mobile social media environment

**DOI:** 10.3389/fpsyg.2022.962224

**Published:** 2022-07-26

**Authors:** Xingjie He, Lixiao Zhu, Lin Sun, Linqian Yang

**Affiliations:** ^1^College of Management Science, Chengdu University of Technology, Chengdu, China; ^2^School of Marxism, Chengdu University of Technology, Chengdu, China; ^3^School of Business, Chengdu University of Technology, Chengdu, China; ^4^College of Communication Science and Art, Chengdu University of Technology, Chengdu, China

**Keywords:** mobile social media environment, brand marketing, consumer emotion, urban economy, emotional marketing

## Abstract

With the development of urban economy and the enhancement of competition among cities, urban marketing has attracted more and more attention. Emotional marketing is a people-oriented marketing strategy, which cannot be ignored under the current economic development and urban development level. Today, with abundant commodities and diversified shopping channels, how to attract new customers, maintain old customers and enhance customer loyalty through emotional marketing has become the focus of enterprises’ work. This paper studies from the perspective of clothing. Facing the fierce market competition, in the marketing era of domestic and foreign big enterprises seeking development by brands, if small and medium-sized enterprises want to survive and develop, they must set up the lofty goal of becoming big enterprises, implement brand marketing, and constantly grow and grow healthily in the process of building strong brands. It can be seen from the research in this paper that the recommendation success of this algorithm is 19% better than that of the traditional algorithm in the case of a certain number of partitions, and it is suitable for being put into extensive practice.

## Introduction

With the economic development and social progress, people’s consumption concepts, attitudes and habits have changed significantly. Nowadays, consumers not only pay attention to the functional value of products or services, but also pay more attention to whether their psychological needs and emotional preferences are met in the process of purchasing and consuming products or services (Stylidis et al., 2020). How to improve the brand equity of enterprises has become a joint research topic of academic and business circles. Scholars have found in related research that enterprises can only gain more markets and lasting competitiveness if they really think about problems from the perspective of consumers and look at the value of services and products provided by enterprises from the perspective of consumers (Rauschendorfer et al., 2022). This value is not determined by the enterprise, but by the consumer’s perception. With the improvement of China’s urbanization level, the number and population of cities are constantly rising, and the competition among cities in talent, capital, science and technology is increasingly obvious (Bolton et al., 2022). In this environment, in order to improve the city’s competitiveness and promote the city’s better and faster development, the concept of city marketing came into being and received wide attention (Artacho et al., 2019). At present, the social and economic development makes the mass consumption demand upgrade, and whether a brand can be recognized by consumers will directly affect the marketing effect and development potential of enterprises. Emotional marketing has become an important means to help enterprises win word of mouth and enhance brand recognition and satisfaction (Leonidou et al., 2021). By purchasing products, consumers enjoy the added value of products and have emotional resonance, thus driving the sales of products (Kim et al., 2020). The analysis of consumer behavior divides consumption into quantitative consumption stage, qualitative consumption stage and perceptual consumption stage. According to modern psychological research, emotion is an important factor that affects people’s acceptance of information. In the era of emotional consumption, what consumers value in purchasing goods is not the quantity, quality and price of goods, but a kind of emotional satisfaction and psychological recognition. Nowadays, the marketing environment has entered the emotional marketing stage. What consumers value when purchasing goods is not the functional utility of the product itself, but a kind of emotional satisfaction and spiritual recognition (Boeuf, 2020). Therefore, any brand needs to endow its products with emotional color from the emotional appeal of consumers, and achieve the ultimate goal of expanding market share and gaining competitive advantage by digging deep into consumers’ inner emotional appeal. When there is no special innovation in products or services, emotion becomes the key factor for consumers to make purchasing decisions (Rietveld et al., 2020). Emotion marketing captures the special emotional needs of consumers, implements the emotional marketing strategy of enterprises, and runs the main thread of “emotion” through the whole process of marketing activities. Emotion marketing starts from consumers’ emotional needs, arouses and arouses consumers’ emotional needs, induces consumers’ spiritual resonance, and integrates emotions into marketing, so that sentimental marketing can win ruthless competition (Marinao-Artigas and Barajas-Portas, 2021). And the brand can make consumers feel more like buying, and it’s easier to turn consumers into loyal customers. Emotional marketing strategy design from the perspective of brand identity can help enterprises to carry out marketing activities in line with consumers’ psychology, effectively improve brand awareness and market influence, and help enterprises achieve long-term development goals. Choosing the right marketing strategy is the guarantee for the success of city marketing. The so-called city marketing strategy refers to the methods and means of developing, packaging, publicizing and selling the city according to the city’s own positioning and development direction, in order to achieve the goal of most effectively attracting target customers and enhancing the overall competitiveness of the city. Among them, emotional marketing strategy is an essential marketing strategy for the development of modern cities. With the economic development, the increase of consumers’ purchasing power, the upgrading of consumption, and the appearance of different products with similar functions in the market, consumers tend to choose products according to their own preferences and values, paying attention to emotional satisfaction and psychological recognition, so that enterprises can gain higher brand loyalty (Faralli et al., 2020). The so-called emotional marketing refers to a marketing method that wins the trust and preference of consumers through psychological communication and emotional communication, thus expanding market share and gaining competitive advantage. Enterprises must fully realize this new change of consumers, actively explore effective emotional marketing methods in marketing, and narrow the emotional distance between enterprises and consumers, so as to realize the integration and high efficiency of product production and marketing.

The innovation of this paper lies in attracting new customers, maintaining old customers and improving customer loyalty through emotional marketing. This paper studies from the perspective of clothing. This paper mainly expounds that small and medium-sized enterprises must implement brand marketing strategy in order to become bigger and stronger in the face of fierce market competition. Brand is the most powerful guarantee for the sustainable development of enterprises. Discuss marketing methods. In the context of the socialized application of Internet technology, enterprises should face the changes in the consumer market and deeply analyze consumer psychology. This paper combines the association rule algorithm to ensure that effective emotional marketing strategies are adopted to encourage consumers to have emotional resonance, so as to improve the marketing effect. It can be seen from the research in this paper that under a certain number of partitions, the recommendation success rate of this algorithm is 19% higher than that of the traditional algorithm, which is suitable for popularization and application.

This paper is divided into five parts: the first part is the background and brief description. This paper expounds that the development of social economy makes the mass consumption demand upgrade, and whether a brand can be recognized by consumers will directly affect the marketing effect and development potential of enterprises. The second part is the related work of this paper. For small and medium-sized enterprises, the most important thing is to solve the survival problem, and brands often show their effectiveness in the further development of enterprises. The third part expounds some important related knowledge and theories of clothing brands and brand marketing. The fourth part discusses the marketing methods and explains some research results and data. The fifth part is the conclusion.

## Related work

Good products and services support the brand. Brand is a multifaceted system, which runs through the strategic development and management of small and medium-sized enterprises. Like products or services, brands also need marketing. It has their own unique rules (Kachen and Krishen, 2020). Due to the inherent defects, lack of resources and capabilities, Mingione SMEs are still relatively slow in brand awareness and brand marketing, and at present, they have not found a completely feasible way of brand marketing, which needs attention (Mingione et al., 2019). Efrat’s labor cost is no longer in a competitive position. The establishment of foundries in other third world countries and regions in the world has brought great difficulties to enterprises’ exports, and the OEM industry model has no sustainable development capability (Efrat and Asseraf, 2019). Hollebeek’s comfortable “no-brand thought” will be extremely dangerous. The process of social development is different, and various values are facing fierce conflict and integration. How to establish brands, famous brands and even strong brands in such a social environment is the concern of every business owner and operator who is interested in market economy (Hollebeek and Macky, 2019). Teichert for small and medium-sized enterprises, the most important thing is to solve the survival problem, and the brand often shows its efficacy in the further development of enterprises (Teichert et al., 2018). Mende brand has formed the intangible assets of enterprises. By owning the intangible assets of brands, enterprises can maximize interest rates and obtain high “added value.” Although brands win by quality, they usually have cultural and emotional connotations, so brands add added value to products and form product differentiation (Mende et al., 2019). Due to the limitation of capital and strength, small and medium-sized enterprises in Yang can’t make their brands bigger in the short term. Instead of choking to death in the jungle where they are struggling to build their brands, they might as well give up their brands, sink their hearts and go all out to make their own products, be vassals or shadows of strong brands to survive first, and then develop their brands when they have strength (Yang and Yen, 2018). Brand Kim is supported by good products and services. Brand is a multi-faceted system that runs through the strategic development and management of small and medium-sized enterprises. Like products or services, brand needs to be marketed, and it has its own unique rules (Kim and Thapa, 2018). Deb brand marketing is actually a kind of marketing behavior of enterprises, which finds out the demands of different customer groups on products and brands through precise market research, establishes the value of related products and brands, and finally meets the needs of customers (Deb and Lomo-David, 2021). Kuanr small and medium-sized enterprises should pay attention to brand and brand marketing at the beginning of their business. On the premise of maintaining a good reputation, they constantly use the same brand to launch cheap and high-quality products. After long-term efforts, they persistently meet the needs of consumers and finally form their own well-known brands (Kuanr et al., 2022). If Smith enterprise is eager to become a big enterprise in the future, with stable sales and profits, and stand like a big international brand, we must attach importance to brand and brand marketing as we have always attached importance to products (Smith and Rose, 2020).

Small and medium-sized enterprises have greatly promoted the development of the national economy, so it is an inevitable choice for the sustainable development of social economy to improve the competitiveness of small and medium-sized enterprises and promote their development. However, according to statistics, China’s small and medium-sized enterprises are facing many problems in their development, the most obvious of which is the short life cycle of small and medium-sized enterprises. Then, in the face of such difficulties and increasingly competitive domestic and international market environment, how should SMEs implement brand marketing is an effective way to improve their competitiveness. Small and medium-sized enterprises must reasonably implement brand marketing strategy, make their products and marketing more personalized and core competitive, and produce emotional marketing for consumers, so as to win the opportunity of development and success. Small and medium-sized enterprises are weak in brand marketing, so it is difficult to coordinate the contradiction between survival and development from a strategic point of view, thus making small and medium-sized enterprises lack core competitiveness. The construction of the core competitiveness of enterprises is a long-term and arduous task for the competitiveness of Chinese enterprises. It is on this basis that the research in this field is carried out in this paper, which is of great significance. Improving the competitiveness of small and medium-sized enterprises and promoting the development of small and medium-sized enterprises is an inevitable choice for the sustainable development of social economy. Brand marketing can be implemented to improve the competitiveness of small enterprises. Through meeting various social needs, absorbing labor force for employment, developing new products, promoting national economic development, etc. The identifiability of brands created by small and medium-sized enterprises and the recognition degree of consumers are very high, and the differences are obvious.

## Related theories

### Brand marketing

As social competition tends to be integrated, regionalized and globalized, cities-as “enterprise groups” in a certain extended sense-are bound to get involved in this competition. After the brand marketing of enterprises, the brand marketing of central cities has been paid more and more attention, which has become an inevitable requirement for the further development of cities in China, and will be used as an important competitive means to promote the overall progress of central cities. In the era of brand competition, China’s small and medium-sized enterprises are facing fierce competition from domestic and foreign brands, and their development is more and more restricted by brands, which has become the key factor restricting the development and growth of small and medium-sized enterprises. Therefore, small and medium-sized enterprises must effectively implement brand marketing strategies in order to gain a firm foothold in the brand battle under the market economy. Effective marketing strategy has become an important measure to promote the development and competitive advantage of small and medium-sized enterprises, and brand marketing is one of the important contents of marketing. Implementing effective brand marketing under the background of big data can enhance the market popularity and competitive advantage of small and medium-sized enterprises, and at the same time reduce marketing costs and risks. The core of brand marketing is brand. In the implementation of brand marketing, marketing activities all revolve around the creation of high value-added brands. Educating and communicating with consumers is the key to creating brand equity. In the short run, this marketing method can promote the short-term sales of products and increase the profits of enterprises. In the long run, this marketing method can strengthen the brand of enterprises and products, deepen the recognition of enterprises and brands in the eyes of consumers, and establish the long-term competitive advantage of enterprises. Word-of-mouth communication actually has “Matthew effect,” which will make leading brands get better communication. For example, someone talks to a stranger in the automobile industry that a brand is the number one automobile in the world. In a flash, the most important brand image of the brand, such as the number one, the best, the most expensive and the most famous in the world, has been rooted in the consumer’s mind. The definition of small and medium-sized enterprises in China refers to enterprises with various forms of ownership, which are legally established in China, and whose operating income, asset scale and employees are within a certain standard range. According to different industries, they have different classification standards. However, no matter what kind of industry SMEs are in, their asset scale and operating income are within a certain standard range. China’s small and medium-sized enterprises occupy an important position in the national economy and play an increasingly important role in economic development. The healthy growth of small and medium-sized enterprises is of great strategic significance to the development of the whole national economy. With the globalization of economy and China’s entry into the WTO, the opening degree of domestic market has been continuously improved, and the pattern and characteristics of market competition are also undergoing new changes. The market competition is intensifying day by day, and the competition among enterprises moves from products to brands. Marketing is characterized by brand marketing as the leading factor, and the establishment of brands is the common choice of all enterprises seeking development. Enterprises in various countries have adopted brand strategies to build strong brands and win the competition. With the explosion of information, the proliferation of advertisements and the busy life, which brands will consumers pay attention to or are willing to pay attention to? Everything can be manufactured, culture can be manufactured in batches, false and exaggerated advertising can be manufactured, corporate images and moving cases can also be planned and manufactured, and scams of brand awards are frequently staged grandly. There is no certified “dental prevention group” that certifies toothpaste brands everywhere. A brand is a title, logo, symbol or design, and its combination and application. Its intention is to facilitate consumers to clearly identify a product or service and distinguish it from the same type of products or services. Many small and medium-sized enterprises still have problems in brand marketing, such as backward brand marketing concept, lack of long-term planning, insufficient application of information technology, and lack of professional brand marketing talents, which limit the effectiveness of brand marketing of small and medium-sized enterprises. Therefore, effective measures should be taken to improve the brand marketing strategy of SMEs and promote their healthy and long-term development under the background of big data. The key difference between brand marketing and traditional marketing concept lies in the change in marketing. It needs to realize the shift of marketing focus from enterprises to consumers. The application of various marketing combination elements must be changed from the traditional 4P (product, price, channel, and promotion) to 4C (meeting demand, reducing cost, providing convenience, and reaching communication), and the marketing mode oriented by market demand should be changed into the marketing mode oriented by consumers, aiming at the greatest satisfaction of consumers. While we are trying to sell products, it is also affecting the growth of enterprises and brand building. Under the above circumstances, how can we achieve this multiple and unavoidable unique effect? Therefore, I think that we should introduce a feedback evaluation and adjustment link in the marketing behavior, so as to ensure that our marketing behavior can better serve products and enterprises, help build brands and promote the healthy development of enterprises, instead of short-lived incentive noise. However, at present, the important topic of city brand marketing is rarely involved in the theoretical circle, which has become the blind spot of marketing theory, which is undoubtedly a great defect and regret. Facing the fierce market competition, in the marketing era of domestic and foreign big enterprises seeking development by brands, if small and medium-sized enterprises want to survive and develop, they must set up the lofty goal of becoming big enterprises, implement brand marketing, and constantly grow and grow healthily in the process of building strong brands.

### Clothing brand

Clothing is known as the symbol of the times. Clothing is a unique perspective to observe social changes. Clothing is the second skin of human beings, and it is also the embodiment of the wearer’s personality. According to legend, ancient China has long been known as the Kingdom of Clothes. About 20,000 years ago, Peking Man who lived in Zhoukoudian cave had already used bone needles, and tubular bone needles were also unearthed at the Neolithic site in Hemudu, Ningbo. The phrase “Yellow Emperor Yao Shun hangs his clothes and the world rules” is regarded by later generations as the source of clothing, while the plain yarn single clothes unearthed in Mawangdui weigh less than 50 g, which makes all modern skilled craftsmen drown. In the twenty-first century, the concept of clothing has changed a lot from before, from the basic function of clothing to the status of today’s show, from catching up with the trend to publicizing individuality and embodying new culture, the role of clothing has changed essentially. Brand personality refers to the brand’s human characteristics and consumers’ perception of this characteristic in the process of external communication. Personality is the core and soul of brand image, and most successful brands consistently pass on their personality to consumers. At present, China’s clothing occupies 1/5 of the world’s clothing market, spreading all over the world and becoming the leader of the world’s clothing production. China is a well-deserved clothing production country in the world. However, the overall processing and management level is not high. The main problem is that middle and low-grade products account for a large proportion, while high value-added products account for a small proportion.

From the aspect of export, although China is the largest clothing exporter in the world, there are no famous Chinese brands in the international market. Looking at the development history of domestic clothing manufacturers, although many well-known domestic brands have been built, there is still no clothing brand that dominates the world. How to build the competitiveness of clothing brands is an urgent research topic in China’s clothing industry. Quite a number of Chinese products can only be exported with other people’s brands. Enterprises only earn meager processing fees with low-level labor, and high additional profits are obtained by foreign investors. In the long run, Chinese garment enterprises will lose their ability to grasp the fashion trends in the international market, and they will be more subject to others. Such disparity makes Chinese garment industry call for the creation of brands. China is a weak brand country, lacking internationally renowned brands, which is out of proportion with China’s status as a big clothing country.

According to statistics, at present, there are about 400,000 clothing brands and thousands of well-known enterprises in China, but their international popularity and reputation are not good. Among the top 100 Chinese brands, there are only two clothing enterprises. The status of clothing brands in China is such that their international competitiveness can be imagined. It wasn’t until the end of nineteenth century that modern garment industry started in China. Chinese garment industry originated from three schools: Benbang, Baibang, and Hongbang. Those who specialize in Chinese gown and pants are called Benbang tailors, those who specialize in fancy cheongsam and women’s clothes are called Baibang tailors, and those who are good at making suits are called Hongbang tailors. Chinese garment industry originated from the latter. The reasons are as follows: firstly, China’s garment enterprises are still in the stage of capital accumulation, and their own financial strength is insufficient. It costs at least 100 million dollars to establish a brand in the United States, which is almost unaffordable for all domestic garment enterprises; Second, it is difficult for foreign consumers to recognize fashion brands from China. Taking suits as an example, it is difficult for Europeans in the birthplace of suits to accept the brand of suits from the “mysterious” East; Third, the design strength of Chinese garment enterprises is insufficient, mainly based on imitation and reproduction, and the original design is obviously insufficient; Fourth, the cultural connotation of Chinese clothing brands is not deep enough, and there is no good integration between national clothing culture and modern clothing; Fifth, China’s trademark of origin has set up obstacles for clothing brands to enter the top international brands.

With China becoming the world’s largest clothing kingdom for clothing production and export, the concept of brand gradually emerged in 1980s with the implementation of famous brand strategy, famous teacher project and famous city project of Chinese clothing. At that time, although the clothing industry had a chain effect on brands, it believed that brands were a good thing. The lack of well-known clothing brands in the world has always been the hidden pain of China’s clothing industry. Because of this, China is a big country in the world in clothing production, but it can’t be called a world clothing power. To become a clothing power, a number of well-known brands must be supported. The methods of brand building include: developing brand personality by new products, building brand personality by brand marketing idea, building brand personality by brand culture and design idea. As the “darling” of fashion, the clothing industry practitioners need to highlight their brand personality among many competitors, especially for the developing original clothing brands. Clothing brand design must pay attention to aesthetic design principles and show the core idea of the brand. Colors, graphics and fonts should be matched reasonably. In addition, we must pay attention to show the core idea of the brand, and pay attention to the market positioning and aesthetics of consumer groups. Only after these basic standards are determined can there be a clear theme direction in the design. The best brand is the guarantee of quality, so once the brand is confirmed, the products under its image will be recognized by consumers and win a good market.

With the gradual maturity of market economy, the brand image of commodities has become the first factor of consumers’ cognition, and the brand effect of commodities has a far-reaching influence in marketing, which has brought huge extra-value profits to businesses. The value of brand, an intangible asset, has been quantitatively evaluated as extraordinary in value. It is very difficult for Chinese garment enterprises to carry out brand strategy in the international market, and they do not have very favorable conditions at present. Therefore, the implementation of brand strategy should focus on the domestic market and neighboring countries, give full play to the characteristics of Chinese national costume culture, start with new materials, new processes, new designs and new styles, and change imitation into innovation. First of all, it will be stronger in China, and gradually implement brand strategy in neighboring countries.

Social media platform can attract users to participate widely and has strong interactivity. Moreover, users can find like-minded netizens to form a community according to their own interests, or establish a network of acquaintances through acquaintances, which has laid a foundation for enterprises to carry out marketing on social media platforms. Social media marketing is a marketing promotion activity based on social media platform. It is different from the traditional marketing activities aimed at selling goods. It hopes to attract users’ attention to the brand, get users’ recognition of the brand, and finally get the brand premium. In the social media environment, enterprises pay more attention to user participation and brand reputation, and hope to establish and maintain a good image of the brand in the hearts of users.

## Research on the influence of brand marketing on consumers’ emotion

### Model building

**FIGURE 1:**
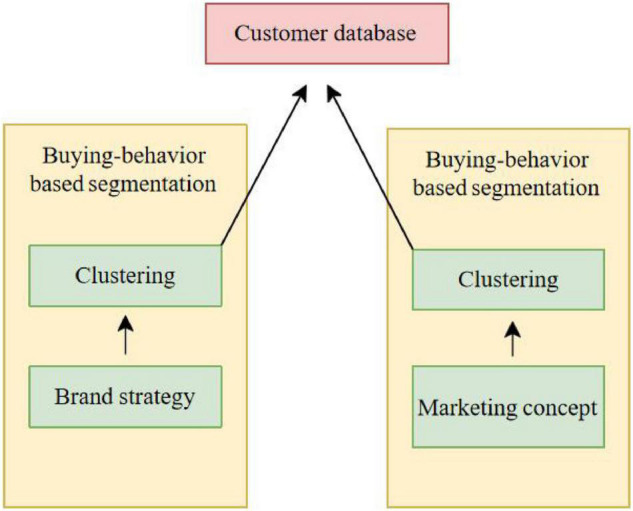
Two types of input data that form user’s interest preferences.

“Economic base determines superstructure” is one of the well-known principles of Marxist philosophy, which is also applicable in the development of marketing. In the early times of agricultural economy and industrial economy, the focus of marketing was basically around the product itself. Because of the low level of economic development at that time, the level of enterprises’ understanding of the delivered value of consumers was not high, and it mainly stayed in the basic functions and utility of products. Two types of input data that form user interest preferences are shown in Figure 1.

With the development and progress of economy, the era of service economy has gradually entered, and the value delivered by consumers has been extended to the service field. By enjoying services, consumers not only feel the value, but also feel the comfort of life, respect and value identity. The so-called emotional marketing refers to a marketing method to win consumers’ trust and preference through the research on consumers’ psychology and emotional communication, so as to expand market share and gain competitive advantage. The other is to regard the emotional differences and needs of consumers as the core of the corporate brand marketing strategy, and achieve the business objectives of the enterprise through the strategies of emotional products, emotional brands, emotional prices, emotional promotion, emotional environment, emotional services, etc.

This paper establishes a similarity matching model based on emotional preference marketing. According to the similarity between the nearest neighbor user and the target user, the weighted sum of the project scores is performed, and the weighted value is used as the predicted score of the target user for the project. Let the user’s estimated score for the item equal to the user’s average score plus the weighted average score of the user’s adjacent users.

Let the estimated score of item *i* by user *u* be equal to the average score of user *u* plus the weighted average score of neighboring users of user *u*.

Where the weight *P_(u, v)* is the normalized value of *SIM*(*u*,*v*):


(1) $${\hat{r}}_{u\,i}={\bar{r}}_{u}+\underset{v\in N\,\left(u\right)}{\Sigma }P_{\left(u,v\right)}\,\left(r_{v\,i}-{\bar{r}}_{v}\right)$$

(2) $$P_{\left(u,v\right)}=\frac{S\,I\,M\,\left(u,v\right)}{\underset{v\in N\,\left(u\right)}{\Sigma }\left|S\,I\,M\,\left(u,v\right)\right|}$$

Since each user’s scoring prediction estimate for each unrated item can be expressed by formula (1), the matrix form of scoring prediction estimate can be derived, as shown in formulas (2) and (3):


(3) $$\overset{⌢}{R}=\bar{R}+P\bar{R}=\bar{R}+P\left(R-\bar{R}\right)$$

(4) $$\overset{⌢}{R}-\bar{R}=P\,\left(R-\bar{R}\right)$$

Emotional marketing is a marketing method that wins consumers’ trust and preference through psychological communication and emotional communication, and then expands market share and gains competitive advantage. That is, enterprises regard consumers’ personal emotional differences and needs as the emotional marketing core of enterprise brand marketing strategy, and achieve enterprise marketing objectives by means of emotional packaging, emotional promotion, emotional advertising, emotional word of mouth and emotional design. Under the background of socialized application of Internet technology, enterprises should face the changes of consumer market, conduct in-depth analysis on consumers’ psychology, and ensure that effective emotional marketing strategy can be adopted to encourage consumers to have emotional resonance and meet their emotional needs, so as to improve marketing effectiveness.

Assuming that the number of rating resources of user *u*_*i*_ in the test set is *N*_*i*_, the rating set of predicted user *u*_*i*_ is *P*_*ij*_(*j* = 1, 2, …, *N*), the actual rating is *q*_*ij*_(*j* = 1, 2, …, *N*_*i*_), and the actual rating set of user *u*_*i*_ is *R*_*max*_, the average absolute deviation *MAE* for user *u*_*i*_ is defined as:


(5) MAE=iΣj=1Ni|pi⁢j-qi⁢j|Ni

Then, *MAE* such as *M* that get the predicted score are the number of users:


(6) $$M\,A\,E=\frac{\overset{N_{i}}{\underset{j=1}{\Sigma }}M\,A\,E_{i}}{M}$$

The average absolute error (*NMAE*) difference of normalization is:


(7) $$N\,M\,A\,E=\frac{M\,A\,E}{R_{\max}-R_{\min}}$$

Emotion is an important part of human psychological activities, and it is the embodiment of people’s inner positive or negative attitude toward external things. In the urban area, emotional marketing is a marketing strategy based on human’s constantly strengthened emotional factors, with the humanistic spirit as the core, and by tapping people’s deep psychological needs, using emotional factors to attract and influence to win the target consumers. Its essence is people-oriented and winning with emotion. Internet communication has the characteristics of strong time domain, high interaction and low cost. Planners keep up with social hotspots and plan and carry out activities according to consumers’ psychological needs.

Let *P*_*u_i_ i_t_*_ represent the predicted score of the target user *u*_*i*_ on the item *i*_*t*_, and *K* is the size of the nearest neighbor set 555.

Finding correlation similarity, also known as Pearson correlation, calculates the linear correlation between user rating vectors to test the similarity between users. The formula is as follows:


(8) s⁢i⁢m⁢(ui,uj)=∑it∈Iu⁢iti⁢(ru⁢itj-r¯ui)⁢(rujit-r¯uj)∑it∈Iu⁢uji(ru⁢iti-r¯ui)2⁢∑it∈Iu⁢uji(ru⁢iji-r¯uj)2

Simply take the average score of the nearest neighbor users for the project as the recommended prediction value of the target users for the project, and the formula is as follows:


(9) $$P_{u_{i}\,i_{t}}={\frac{1}{K}}^{*}\,\underset{u_{j}\in N}{\Sigma }r_{u_{j}\,i_{t}}$$

According to the similarity between the nearest neighbor user and the target user, the score value of the project is weighted and summed, and the weighted value is used as the target user’s predicted score for the project. The formula is as follows:


(10) Pu⁢iti=Σuj∈Ns⁢i⁢m⁢(ui⋅uj)uj⁢it*Σuj∈Ns⁢i⁢m⁢(ui⋅uj)

Average standardized calculation is adopted to eliminate the influence of user evaluation scale on recommendation, and the formula is as follows:


(11) Pui⁢it=r¯ui+Σuj∈Ns⁢i⁢m⁢(ui⋅uj)*⁢(ruj⁢it-r¯uj)Σuj∈Nsim(ui⋅u)j

**FIGURE 2:**
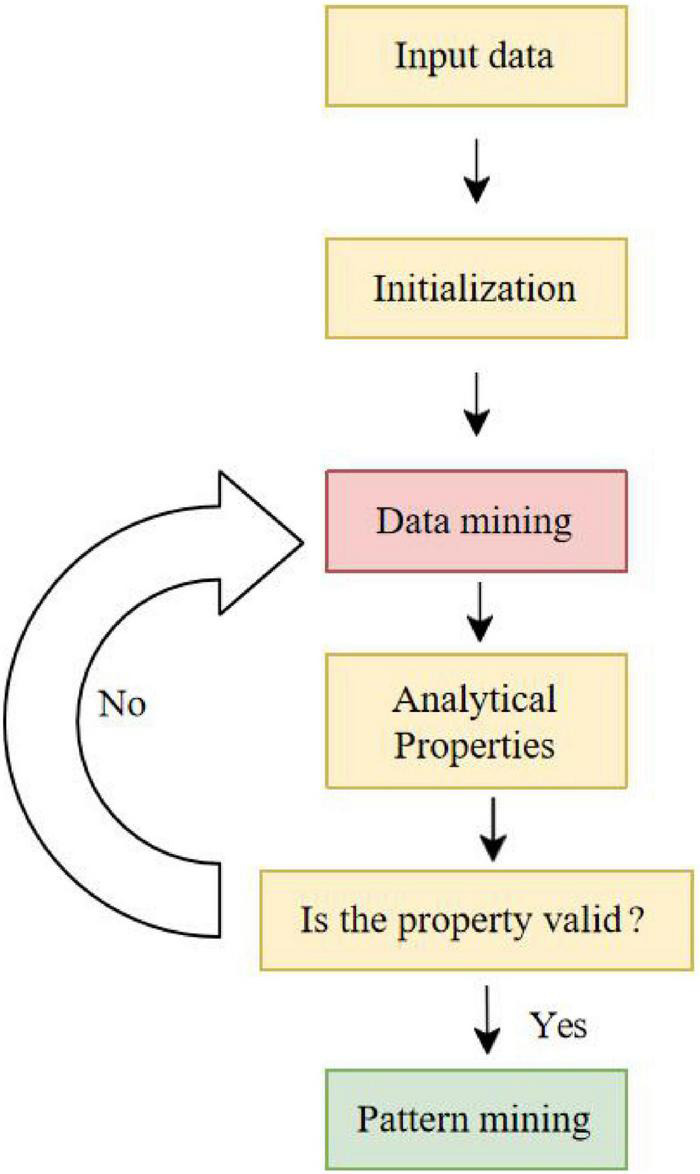
Flow chart of algorithm implementation.

With the arrival of the information age, the material products are greatly enriched, and the competition is intense. The overall level and composition of people’s consumption are developing toward high-level, comfortable, and emotional, and people’s consumption needs are becoming increasingly differentiated, diversified, personalized, complicated, and emotional. With the further development of economy, marketing experts put forward the concept of experience economy, and think that experience is the economic product of this stage. At this stage, the delivered value of consumers has been further recognized and developed, and consumers can get psychological pleasure through personalized and humanized experience. This kind of consumer delivered value developed by the emotional feelings gained by consumers has prompted emotional marketing to emerge as the times require and become the marketing theme in the new economic era. The flow chart of algorithm implementation is shown in Figure 2.

In the era of emotional satisfaction, many people of insight in the business world began to notice that if emotional elements were injected into marketing tools to make marketing more emotional, the original hard marketing could be turned into humanized marketing with modern charm, which would capture consumers’ hearts and make consumers have emotional loyalty to brands. This is the direct cause of emotional marketing. People pay more attention to the publicity of personality and the pleasure of spirit, and the consumption concept of “emotional value” becomes more and more obvious, while the consumption concept of “functional value” becomes weaker and weaker. Emotional marketing has become a new sharp weapon to establish corporate image, enhance brand reputation, expand market, consolidate and develop customer relationship, and its influence is increasingly significant and far-reaching.

Marketing endows goods with spiritual connotation and spirituality in packaging, design, service and other related aspects, which will make consumers strongly infected and impacted, stimulate consumers’ potential purchasing consciousness and desire, and gradually expand the market share of products, so that enterprises can get rich returns. Fundamentally speaking, the key to the prosperity and prosperity of a city lies in people’s efforts and changes. The focus of urban construction and development is to put people in the first place and care for and consider people’s basic needs. The emotional marketing of the city is to show the city’s comprehensive environment, including the city’s natural environment, living environment and employment environment, which reflect the city’s own characteristics, to the target customers, that is, the city’s citizens, investors and tourists, so that they can feel the city’s service concept of being convenient, caring for people and benefiting people, and their spiritual care under the pressure of high-intensity work rhythm and competition, thus making them have emotional preference and dependence, making the city gain an advantage in the fierce competition, and finally winning consumers. Get in touch with consumers from visual, auditory and other dimensions, get feedback from consumers while causing discussion, and adjust the activity plan in time according to consumers’ opinions, resulting in the effect that one plus one is greater than two.

### Result analysis

When recommending a new package to corporate customers, the usual practice is to send the information of the package to as many mobile phone users as possible. Once the mobile phone users receive this advertising message, they will decide whether to customize the package according to their own wishes and consumption characteristics. Traditional collaborative filtering recommendation algorithm has been widely used because of its simplicity and practicality, but there are many problems, especially in the similarity calculation. The core part of collaborative filtering recommendation algorithm is the similarity calculation of users or items. However, for a long time, the research on collaborative filtering recommendation algorithm is mostly based on the direct calculation of similarity, which has many defects. Because the similarity between projects is relatively stable, if there is no direct similarity, it can also be calculated by the attributes of the projects, so that data sparseness will not have a great impact on the similarity calculation.

**FIGURE 3:**
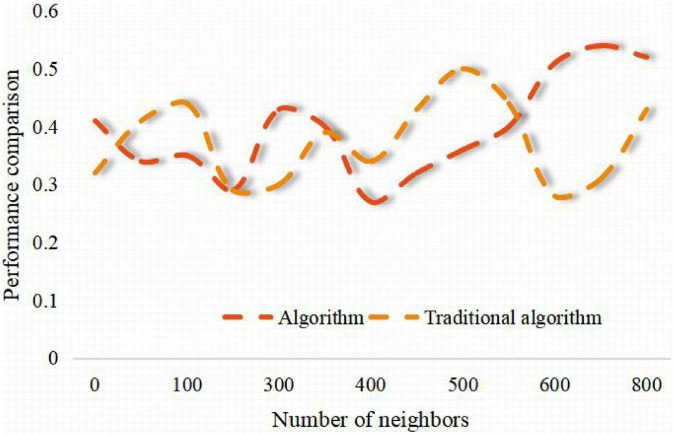
Performance comparison chart.

**FIGURE 4:**
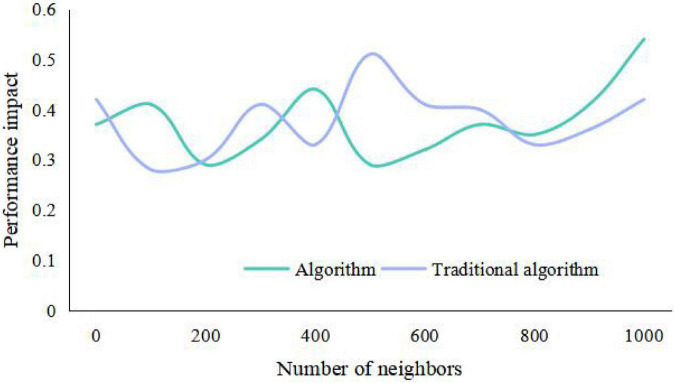
Influence of support weight on algorithm performance.

**FIGURE 5:**
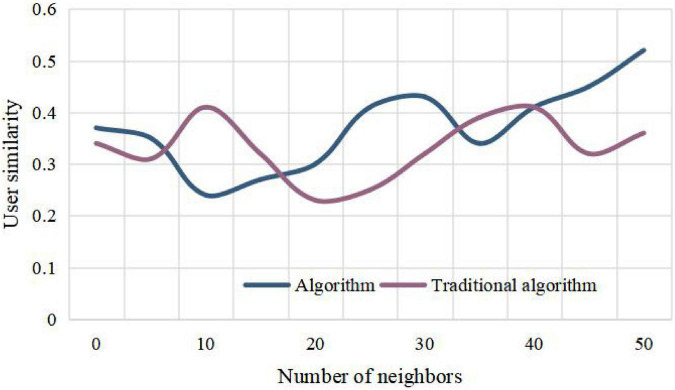
Comparison of user similarity calculation methods under training set.

**FIGURE 6:**
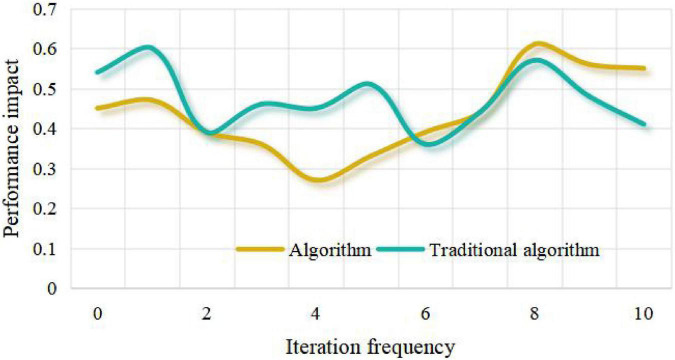
Influence of iteration frequency on the performance of similarity learning algorithm without considering fitting.

Inspired by this, we get a way to solve the sparseness of collaborative filtering data based on users, that is, predict and score some unrated items through the links between items, so as to alleviate the sparseness of data. The research adopts the brand marketing data of some small and medium-sized enterprises in the market. In the process of building a strong brand, this paper analyzes this article. According to the similarity between the nearest neighbor user and the target user, the weighted sum of the item scores is performed, and the weighted value is used as the target user’s predicted score for the item. It can be seen from the research of this paper that the recommendation success rate of this algorithm is higher than that of the traditional algorithm under a certain number of partitions. From Figures 3–6, it can be seen that the number of iteration steps affects the algorithm based on two-stage similarity learning. It can be seen from the performance comparison chart in Figure 3 that when a large number of customer analysis is required. Compared with the traditional algorithm, this algorithm has high advantages. The level of algorithm competition is much higher than the general algorithm. It can be seen that this algorithm is superior to the traditional algorithm, and the algorithm in this paper can achieve the best performance with a small number of iterations and good error performance.

**FIGURE 7:**
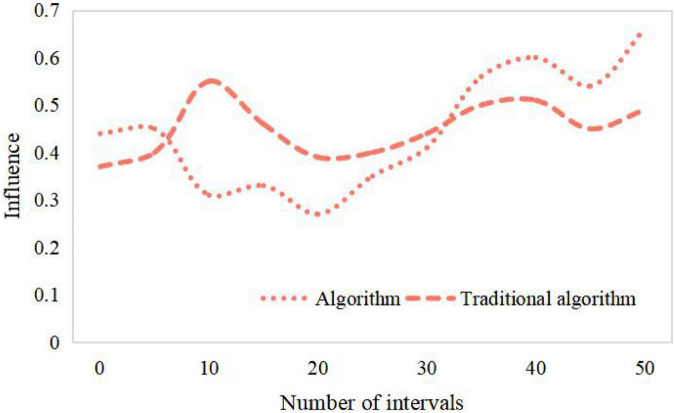
Influence of different number of partition intervals on results.

**FIGURE 8:**
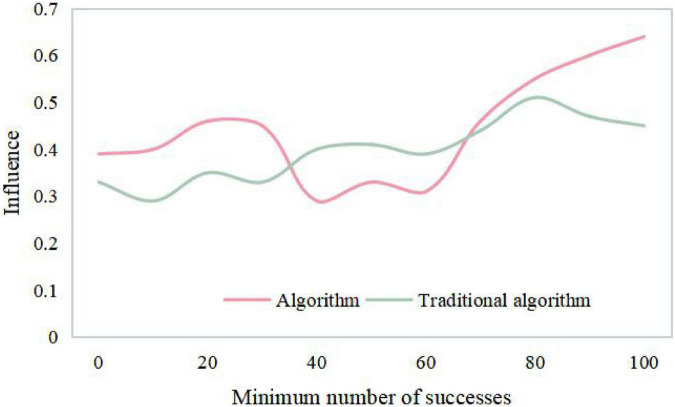
Curve diagram of the influence of different minimum success numbers on the results.

The algorithm collects the information of users’ preference to items, analyzes users’ interests, finds the nearest neighbor users with similar rating bias to the current users, and predicts the unrated items of the current users according to the rating data of the nearest neighbor users. Finally, the items with higher rating or the predicted value greater than the set rating threshold are recommended to the target users, and the branded clothing products will get more user interests and be recommended to users. There is only one recommended product for the enterprise package recommendation data set, that is, each user has a variety of different characteristics to predict whether the user is interested in this product, and the emotional factors generated by the brand also play an important role. Therefore, according to the relevant situation that collaborative filtering technology is suitable for, this data set is not suitable for user recommendation by collaborative filtering technology. From Tables 1, 2 and Figures 7, 8, it can be seen that the recommendation success of this algorithm is 19% better than that of the traditional algorithm with a certain number of partitions. This is because when the number of intervals divided in this paper is fixed, the minimum success requirement value in each interval is larger.

**TABLE 1:** Influence of different number of partition intervals on results.

	0	5	10	15	20	25	30	35	40	45	50
Algorithm	0.44	0.45	0.31	0.33	0.27	0.35	0.41	0.56	0.60	0.54	0.66
Traditional algorithm	0.37	0.40	0.55	0.46	0.39	0.40	0.44	0.50	0.51	0.45	0.49

**TABLE 2:** Curve diagram of the influence of different minimum success numbers on the results.

	0	10	20	30	40	50	60	70	80	90	100
Algorithm	0.39	0.40	0.46	0.45	0.29	0.33	0.31	0.46	0.55	0.60	0.64
Traditional algorithm	0.33	0.29	0.35	0.33	0.40	0.41	0.39	0.44	0.51	0.47	0.45

There is a big deficiency in collaborative filtering based on items. It is impossible to make new and different recommendations depending on the similarity between items, and the recommendation results can only cater to the current interests and preferences of users, which greatly reduces the practical value of predictive scoring. The improved method of data sparsity based on correlation analysis predicts and scores the unrated items not according to the similarity between items, but according to the correlation between items, which not only achieves the purpose of improving data sparsity, but also can make new and different recommendations. Generally, the data source of collaborative filtering algorithm is the user’s display rating information of the project. Of course, users’ browsing and access logs can also be analyzed to quantify the implicit evaluation matrix of the user’s project.

A user item rating matrix is constructed with users as rows and items as columns, and each value in the matrix corresponds to the user’s rating on the item. If the rating is blank, the default value is that the higher the score, the more inclined the user is to the item, otherwise, the lower the user’s interest in the item. Therefore, in order to improve the accuracy of collaborative filtering recommendation, we must first start with the similarity calculation, and we need to find a more reasonable similarity calculation method to improve the accuracy of recommendation. After using this user recommendation method, enterprises can randomly select some users and recommend packages to them, and then they will get feedback data on whether users handle packages. By analyzing these data and training, enterprises can get the consumption characteristics of users who tend to customize the packages. In this way, when the enterprise sends the advertisement recommendation of the package again, it can choose to recommend the package only for all users who meet the corresponding consumption characteristics according to the analyzed data.

Association analysis, the purpose of which is to find out the hidden association between different data items (commodities) in the database. The products of association analysis include frequent patterns and association rules. Frequent patterns are patterns that frequently appear in data sets (such as itemsets, subsequences or substructures, etc.); Association rules are rules that describe the potential relationships among the data in the database. For example, these rules reflect the influence of buying a certain commodity on buying other commodities. Association rules are the result of further mining frequent patterns. The search of the nearest neighbor user set is a key part of collaborative filtering algorithm. If the nearest neighbor search is inaccurate and there is a big difference between the nearest neighbor search and the target user’s interest, there will be scoring bias in the next prediction, which will directly affect the accuracy of recommendation results. Not all users in the user group have a great connection with the target users, so there is no need to learn many user similarity parameters with a small connection degree, and in fact, the users who are really connected with the target users often only account for a small part of this user group, that is, the number of users’ neighbors is far less than the total number of the group. Although the advertisements recommended by packages are only recommended to some users, it seems that the coverage rate is low. However, because this recommendation method is more targeted, the hit rate of package processing will be greatly improved and the number of spam messages will be reduced.

## Conclusion

This paper discusses the importance of brand marketing strategy for small and medium-sized enterprises. If small and medium-sized enterprises want to survive and develop, they must set the lofty goal of becoming big enterprises. Brand marketing strategy must be implemented, and brand is the most powerful guarantee for the sustainable development of enterprises. Implement brand marketing, and keep growing and healthy in the process of building a strong brand. This paper mainly expounds that small and medium-sized enterprises must implement brand marketing strategy in order to become bigger and stronger in the fierce market competition. Brand is the most powerful guarantee for the sustainable development of enterprises. Discuss marketing methods. In the context of the socialized application of Internet technology, enterprises should face the changes in the consumer market and deeply analyze consumer psychology. This paper combines the association rule algorithm to ensure that effective emotional marketing strategies are adopted to encourage consumers to have emotional resonance, so as to improve the marketing effect. It can be seen from the research in this paper that under a certain number of partitions, the recommendation success rate of this algorithm is 19% higher than that of the traditional algorithm, which is suitable for popularization and application.

However, small and medium-sized enterprises usually lack comprehensive, systematic, and long-term brand marketing strategy knowledge, and often make wrong decisions on brand marketing strategy, which will not only make enterprises lose market opportunities, but also make enterprises lose their own reputation and brand value. Therefore, this paper needs to further modify the analysis in the future analysis.

## Data availability statement

The original contributions presented in this study are included in the article/supplementary material, further inquiries can be directed to the corresponding author.

## Author contributions

All authors listed have made a substantial, direct, and intellectual contribution to the work, and approved it for publication.

## Conflict of interest

The authors declare that the research was conducted in the absence of any commercial or financial relationships that could be construed as a potential conflict of interest.

## Publisher’s note

All claims expressed in this article are solely those of the authors and do not necessarily represent those of their affiliated organizations, or those of the publisher, the editors and the reviewers. Any product that may be evaluated in this article, or claim that may be made by its manufacturer, is not guaranteed or endorsed by the publisher.
